# GC-MS-Based Endometabolome Analysis Differentiates Prostate Cancer from Normal Prostate Cells

**DOI:** 10.3390/metabo8010023

**Published:** 2018-03-19

**Authors:** Ana Rita Lima, Ana Margarida Araújo, Joana Pinto, Carmen Jerónimo, Rui Henrique, Maria de Lourdes Bastos, Márcia Carvalho, Paula Guedes de Pinho

**Affiliations:** 1 UCIBIO/REQUIMTE, Department of Biological Sciences, Laboratory of Toxicology, Faculty of Pharmacy, University of Porto, 4050-313 Porto, Portugal; ritacmlima@hotmail.com (A.R.L.); ana.margarida.c.araujo@gmail.com (A.M.A.); jipinto@ff.up.pt (J.P.); mlbastos@ff.up.pt (M.d.L.B.); mcarv@ufp.edu.pt (M.C.); 2 Cancer Biology & Epigenetics Group, Research Center (CI-IPOP) Portuguese Oncology Institute of Porto (IPO Porto), 4200-072 Porto, Portugal; carmenjeronimo@ipoporto.min-saude.pt (C.J.); rmhenrique@icbas.up.pt (R.H.); 3 Department of Pathology and Molecular Immunology-Biomedical Sciences Institute (ICBAS), University of Porto, 4050-313 Porto, Portugal; 4 Department of Pathology, Portuguese Oncology Institute of Porto (IPO Porto), 4200-072 Porto, Portugal; 5 UFP Energy, Environment and Health Research Unit (FP-ENAS), University Fernando Pessoa, 4249-004 Porto, Portugal

**Keywords:** prostate cancer, cell lines, metabolomics, endometabolome, GC-MS

## Abstract

Prostate cancer (PCa) is an important health problem worldwide. Diagnosis and management of PCa is very complex because the detection of serum prostate specific antigen (PSA) has several drawbacks. Metabolomics brings promise for cancer biomarker discovery and for better understanding PCa biochemistry. In this study, a gas chromatography–mass spectrometry (GC-MS) based metabolomic profiling of PCa cell lines was performed. The cell lines include 22RV1 and LNCaP from PCa with androgen receptor (AR) expression, DU145 and PC3 (which lack AR expression), and one normal prostate cell line (PNT2). Regarding the metastatic potential, PC3 is from an adenocarcinoma grade IV with high metastatic potential, DU145 has a moderate metastatic potential, and LNCaP has a low metastatic potential. Using multivariate analysis, alterations in levels of several intracellular metabolites were detected, disclosing the capability of the endometabolome to discriminate all PCa cell lines from the normal prostate cell line. Discriminant metabolites included amino acids, fatty acids, steroids, and sugars. Six stood out for the separation of all the studied PCa cell lines from the normal prostate cell line: ethanolamine, lactic acid, β-Alanine, L-valine, L-leucine, and L-tyrosine.

## 1. Introduction

Cancer is a very relevant health concern worldwide, both in terms of morbidity and mortality [1]. Prostate cancer (PCa) is one of the most prevalent cancers in men [2], and the five-year survival for men with metastatic disease is very low (28%) due to the development of castration-resistance following androgen deprivation therapy, which is the most frequent therapeutic approach used for the treatment of advanced PCa. Nevertheless, PCa has a long latency period and is potentially curable if diagnosed early [1]. 

The most commonly used biomarker of PCa in clinical practice is serum prostate specific antigen (PSA). This biomarker reached the clinical practice as early as the 1990s and revolutionized the management of PCa, leading to an increase in the number of PCa cases diagnosed at the early stages [3], with a consequent decrease in PCa mortality [4]. However, due to the limited sensitivity and specificity of this biomarker [5,6,7] and its inability to discriminate aggressive from indolent PCa, overdiagnosis and overtreatment are current issues. Hence, the use of serum PSA for massive screening tests in men over 55 years old is no longer recommended [8]. As such, the discovery of new biomarkers for PCa diagnosis that surpasses these drawbacks is urgently needed.

After a study published in 2009 which proved the concept that metabolomics have the potential to deliver a new biomarker for PCa with good sensitivity and specificity, the interest of the scientific community in metabolomics applied to PCa markedly increased. In this study, sarcosine was suggested as a potential biomarker for PCa which could be assessed in urine [9]. Taking into consideration the very encouraging results of this study, several other researchers tried to replicate it to confirm the potential of sarcosine as PCa biomarker, but results were contradictory. Whereas some studies confirmed the potential of sarcosine as PCa biomarker [10,11,12,13], others did not observe a significant increase in sarcosine levels in PCa samples compared with controls [14,15,16]. Nevertheless, the potential of metabolomics for biomarker discovery and for understanding PCa biochemistry was proved, and several other studies applied this methodology in several different PCa matrices [17]. 

Metabolomic studies can be performed using several matrices, including biofluids (commonly urine and serum/plasma), tissues, and cell lines [17]. Each of these matrices has advantages and disadvantages. The major advantages of using cell lines in metabolomic studies is that confounding factors like age, concurrent morbidities, diet, smoke habits, and influence from different adjacent tissues are not present. Moreover, problems with interindividual variability are nonexistent. Cell lines are less complex, and the results are easier to interpret and more reproducible when compared with biofluids and tissues from humans or animal models. Also, ethical problems inherent to the use of animal models or human subjects are obviated [18,19,20]. However, cell lines are not able to exactly mimic human cancers because it is practically impossible to simulate complex cell–cell and cell–matrix interactions in cell culture, and these interactions are very important for metabolic alterations which occur during tumor progression [19,21].

The main objective of this study was to prove the concept that a metabolomic profile is able to discriminate PCa cells from normal prostate cells by means of metabolic signatures of different cultured cancer cells, comparing them with a normal prostate epithelium immortalized cell line. Intracellular metabolites which were significantly increased or decreased as a consequence of PCa were also assessed. For this purpose, a gas chromatography–mass spectrometr **y** (GC-MS) based methodology was applied to analyze one normal prostate cell line and four PCa cell lines with different metastatic potential and androgen receptor (AR) expression status.

## 2. Results

In this study, we performed a GC-MS-based untargeted metabolomic approach to evaluate alterations in the intracellular metabolites of PCa immortalized cell lines compared with a normal prostate immortalized cell line. A total of 150 features were detected in the chromatograms, and a multivariate data analysis approach was applied to confirm that the alterations in endometabolome could be discriminated among the cell lines used in this study.

**Figure 1:**
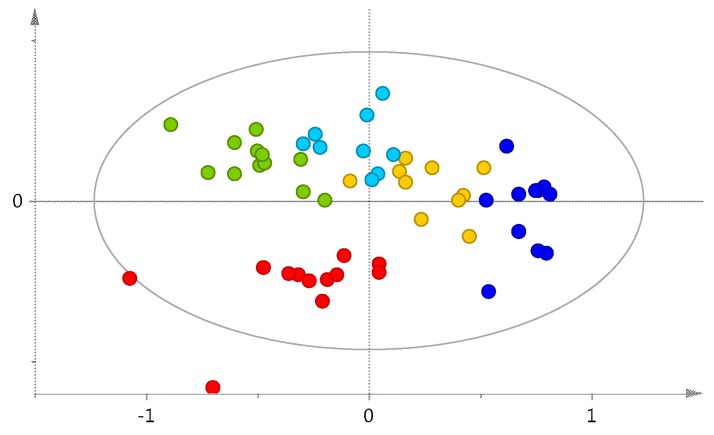
Partial least squares-discriminant analysis (PLS-DA) scores scatter plot (R2X = 0.618; R2Y = 0.386; Q2 = 0.35) with metabolites from all cells lines (PNT2 (green), 22RV1 (dark blue), PC3 (light blue), DU145 (red), and LNCaP (yellow)). It is possible to observe the discriminant capability of the endometabolome analyzed by GC-MS as each cell line forms an independent cluster.

The reproducibility of the analytical method was confirmed by the QCs cluster in a principal component analysis (PCA) model containing all acquired samples (Supplementary Figure S1). Furthermore, multivariate analyses confirmed that a set of metabolites were able to discriminate PCa cell lines from a normal prostate cell line and among the different PCa cell lines. This discriminative trend was observed not only in partial least squares-discriminant analysis (PLS-DA) analysis (Figure 1) but also in PCA (data not shown).

**Figure 2:**
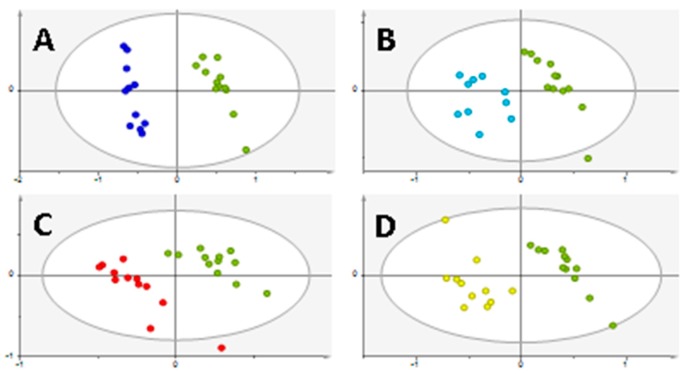
PLS-DA from PCa versus PNT2. (**A**) 22RV1 (dark blue) versus PNT2 (green) (R2X = 0.724; R2Y = 0.978; Q2 = 0.964). (**B**) PC3 (light blue) versus PNT2 (green) (R2X = 0.709; R2Y = 0.887; Q2 = 0.817). (**C**) DU145 (red) versus PNT2 (green) (R2X = 0.722; R2Y = 0.916; Q2 = 0.879). (**D**) LNCaP (yellow) versus PNT2 (green) (R2X = 0.734; R2Y = 0.932; Q2 = 0.894). In all PLS-DA it is possible to observe the discriminant capability of the endometabolome analyzed by GC-MS to differentiate PCa cell lines from the normal cell line, as each cell line forms an independent cluster Q2 > 0.5.

To evaluate which metabolites were responsible for this separation, all cancer cell lines were compared with the normal cell line, namely 22RV1 versus PNT2, PC3 versus PNT2, DU145 versus PNT2, and LNCaP versus PNT2. An optimal separation between each PCa cell line and the normal cell line was observed (Figure 2).

The results of the permutation test showed that all models created were robust for the discrimination between each PCa cell line and the normal cell line (Supplementary Figure S2). 

All metabolites with VIP values higher than one were considered potentially relevant for the separation among the cell lines. Hence, a total of 26 metabolites were considered relevant to differentiate 22RV1 from PNT2, 31 metabolites to differentiate PC3 from PNT2, 37 metabolites to differentiate DU145 from PNT2, and 32 metabolites to differentiate LNCaP from PNT2. After univariate analysis, a total of 18 metabolites were shown to differentiate 22RV1 from PNT2, 18 metabolites to differentiate PC3 from PNT2, 20 metabolites to differentiate DU145 from PNT2, and 23 metabolites to differentiate LNCaP from PNT2 (Table 1). For all these metabolites, the area under the curve (AUC) value was calculated, and the majority of AUC values were very close to one (Table 1). Furthermore, the results obtained revealed that the majority of the discriminant sets displayed a sensitivity and specificity for distinguishing PCa cell lines (22RV1, PC3, DU145, and LNCaP) from the normal prostate cell line (PNT2) of 100% or near this value (confidence interval of 95%) (Table 2).

From all evaluated metabolites, six stand out as important for the separation between all PCa cell lines and the normal cell line: ethanolamine, lactic acid, β-alanine, L-valine, L-leucine, and L-tyrosine. Considering different metabolites, it was also possible to distinguish PCa with different aggressiveness. For example, glycerol was significantly decreased in the low (LNCaP) and the moderate (DU145) metastatic potential cell lines, but not in the highly metastatic cell line (PC3), whereas methyl 2-acetamido-2-deoxy-3-*O*-methyl-a-d-galactopyranoside was significantly increased in DU145 and in LNCaP but not in PC3. The significant increase of 2-butenoic acid levels was a characteristic alteration of the cell line with moderate metastatic potential (DU145); the significant increase of palmitic acid and of 13-octadecenoic acid were characteristic of LNCaP, a cell line with low metastatic potential. It was also possible to discriminate between androgen-responsive and androgen-unresponsive PCa cell lines using urea, since this metabolite was significantly decreased in androgen-unresponsive PCa cell lines (i.e., 22RV1, PC3, and DU145). As previously mentioned, 22RV1 was originally an androgen-receptor-positive cell line, but since the cell culture was performed in the absence of androgen supplementation, we may hypothesize that this cell line developed androgen independency by a mechanism similar to that occurring in clinical practice after hormone deprivation therapy [22].

Several other metabolites identified were also able to discriminate between different cancer cell lines and normal cell lines, namely L-alanine (PC3 versus PNT2, DU145 versus PNT2, and LNCaP versus PNT2), 3-hydroxyisovaleric acid (PC3 versus PNT2, DU145 versus PNT2, and LNCaP versus PNT2), glycine (22RV1 versus PNT2, PC3 versus PNT2, and LNCaP versus PNT2), phenylalanine (22RV1 versus PNT2, PC3 versus PNT2, and LNCaP versus PNT2), sorbose (22RV1 versus PNT2, DU145 versus PNT2, and LNCaP versus PNT2), cholesterol (22RV1 versus PNT2, PC3 versus PNT2, and LNCaP versus PNT2), glutamine (22RV1 versus PNT2 and LNCaP versus PNT2), and palmitoleic acid (22RV1 versus PNT2 and DU145 versus PNT2). Some unidentified metabolites were also important for the discrimination between cancer and normal cell lines (Table 1).

## 3. Discussion

In this study, the potential of endometabolome investigation for discriminating between four PCa cell lines and a normal prostate cell line was explored, indicating that the endometabolome may be a valuable source for PCa biomarkers. Beyond allowing the discrimination between PCa cell lines and a normal prostate cell line, the study of alterations in intracellular metabolites also revealed that it was possible to discriminate among PCa cells with different aggressiveness and it was also possible to discriminate androgen-responsive from androgen-unresponsive cell lines.

The prostate cell has a unique metabolic profile, as one of the major functions of these cells is the production and accumulation of citrate (a component of the prostatic fluid) [23]. Unlike other human cells, the healthy prostate cell does not preferentially use citrate in the Krebs cycle for adenosine triphosphate (ATP) production (aerobic ATP production). Even in the presence of oxygen, the healthy prostate cell produces ATP mainly by glucose oxidation, which leads to pyruvate production and, consequently, to lactate production. However, prostate cells that undergo neoplastic transformation lose their ability to accumulate citrate and use citrate for the Krebs cycle [19,24,25,26]. This well described alteration may explain the significant reduction in the levels of lactate observed in all PCa cells used in this study (Table 1). 

Another possible indication of alterations in the Krebs cycle associated with PCa observed in our study was the significant increase in glutamine levels in PCa cell lines 22RV1 and LNCaP, as well as in PC3, although this was not statistically significant. The conversion of glutamine to α-ketoglutarate, an intermediate of the Krebs cycle, takes place in two reaction steps: (1) The hydrolysis of the amino group of glutamine yielding glutamate, which can be excreted, or (2) may be further metabolized into α-ketoglutarate. Furthermore, glutamine is also important for lipogenesis [27,28], so the significant increase in glutamine levels observed in PCa cells may indicate alteration in lipids and in energetic metabolism of the PCa cells. The alteration in energetic metabolism associated with PCa was also suggested by the significant decrease of sorbose levels since sugars, like sorbose, can be used by cancer cells for energy production. Interestingly, the significant decrease in sorbose levels was previously observed in urine from PCa patients [29]. 

Mucins belong to the glycosylate protein family and the increase in their levels has been associated with cancer, as these compounds are involved in signaling, cell growth, and survival, prompting tumor progression [30]. These important roles of mucins in cancer may explain the significant increase in PCa cells (DU145 and LNCaP), compared with normal prostate cells, in the mucin fragment methyl 2-acetamido-2-deoxy-3-*O*-methyl-a-d-galactopyranoside observed in our study. 

As previously mentioned, the increase in sarcosine levels is one of the most studied metabolic alterations related with PCa and one of its most promising biomarkers [9]. However, the increase in sarcosine levels in PCa is also one of the most controversial metabolic alterations associated with PCa development and progression because several recent studies were unable to confirm this alteration in urine and prostate tissues from PCa patients [14,15,16]. In our study, we were unable to confirm a significant increase of sarcosine levels in PCa cells tested; however, a significant increase in glycine levels was observed in three PCa cell lines: 22RV1, PC3, and LNCaP. Glycine is a precursor of sarcosine. The alteration of this metabolite may also suggest an alteration in the metabolic pathway involved in sarcosine synthesis. However, the alteration of glycine levels may also be associated with dysregulation of other important metabolic pathways as glycine is also an important protein precursor and provides a C2N subunit for purine synthesis [31], a fundamental step for cell proliferation. Thus, this significant increase in glycine levels may be associated with a deregulation in prostate cell proliferation and an active anabolic metabolism in PCa cells [32]. Moreover, glycine is also important for glutathione synthesis, which is involved in the cell defense against oxidative stress. Therefore, the significant alteration in glycine levels may also be related with a high oxidative stress in PCa cells [32].

Beyond glycine, several other amino acids were significantly altered in PCa cells, namely L-alanine, β-alanine, valine, leucine, threonine, phenylalanine, and tyrosine, which may indicate a potential increase in protein synthesis. The increase in protein metabolism is important for cancer cells to maintain an elevated proliferative state as proteins are essential for cell division. The alteration in amino acid levels and, consequently, in protein metabolism was previously found in other PCa metabolomics studies [29,33,34]. Also found was the alteration in β-alanine metabolism, which was also associated with PCa [9].

The urea cycle uses ammonia to produce urea, as urea is less toxic than ammonia. The main source of ammonia is the degradation of proteins and, as previously explained, cancer cells have an increased requirement of proteins which may explain the significantly decreased levels of urea in PCa cells (22RV1, PC3, and DU145) observed in our study. Our results also revealed a significant increase of 3-hydroxyisovaleric acid levels in PCa cells (PC3, DU145, and LNCaP). This leucine metabolite has an important role in protein metabolism once 3-hydroxyisovaleric acid stimulates protein synthesis and prevents protein catabolism [35]. 

The significant elevation in the cholesterol levels observed in PCa cells (22RV1, PC3, and LNCaP) has been previously described in other metabolic studies using prostatic tissue from PCa patients [36,37]. The PCa cells have the ability to synthetize cholesterol. Furthermore, a study performed by Awad et al. (2001) revealed that in vitro supplementation with cholesterol increases cell proliferation, migration, and invasion in PCa cell lines [38]. The relevant role of cholesterol in PCa metabolism may be due to the increased need of membrane biosynthesis in cancer cells and the need of growth factor signaling. Cholesterol may also have an important role in castration resistance of PCa once cholesterol can be converted in vivo to androgens [37].

The alteration in lipid metabolism, particularly in phospholipid membrane constituents’ assembly and catabolism, is frequently associated with PCa [17]. In our study, the alteration in this metabolic pathway is suggested by the significant decrease of ethanolamine levels in all studied PCa cell lines. Ethanolamine is the precursor and the degradation product of phosphatidylethanolamine (an abundant phosphoglyceride in the human cellular membrane), being one of the main components of the cellular membrane. Therefore, the significant reduction of ethanolamine levels in PCa cells suggests an increase in ethanolamine recruitment for membrane cell assembly and, consequently, an alteration in membrane metabolism. The alteration in ethanolamine levels in PCa (serum and tissue) was previously reported by other authors [39,40]. Mintz et al. (2008), using radiolabeled ethanolamine, demonstrated that PCa cells display an increase in ethanolamine uptake compared with normal prostate cells (mostly in androgen-responsive cells in the presence of androgens) [41]. Glycerol is another important metabolite involved in lipid metabolism as glycerol is present in the composition of triglycerides and phospholipids. The catabolism of these lipids to produce energy leads to the formation of glycerol, and glycerol can also be converted into glucose (the principal cellular source of energy). Therefore, the dysregulation in glycerol levels (i.e., a significant decrease in DU145 and LNCaP) is indicative of altered lipid metabolism and of the high-energy demand characteristic of cancer cells to sustain its high proliferation rate [42,43,44]. 

We observed significant alterations in organic acids in PCa cell lines, namely 2-butenoic acid (in DU145) and also the significant increase in the levels of the fatty acid 13-octadecenoic acid (in LNCaP). Similar alterations were associated with PCa in previous metabolomic studies, performed in blood and urine [14,45,46]. Fatty acids may be produced by the β-oxidation of lipids to produce energy. Furthermore, these compounds may also be involved in other cellular mechanisms, namely cell signaling and cell membrane integrity. Thus, the alteration in these metabolites may indicate that cancer cells display altered energetic and lipid metabolism [14,45,46]. In this study, we also observed a significant increase in palmitic acid levels in the LNCaP cell line. The increase of palmitic acid levels in plasma was previously associated with the risk of PCa development in a prospective case-control study [47]. Palmitic acid is produced in vivo from other fatty acids and from de novo lipogenesis, so the significant increase of palmitic acid levels is indicative of altered lipid metabolism, mainly an increase in fatty acid synthesis [47]. Besides palmitic acid, palmitoleic acid is another fatty acid significantly increased in the PCa cells in our study.

**Figure 3:**
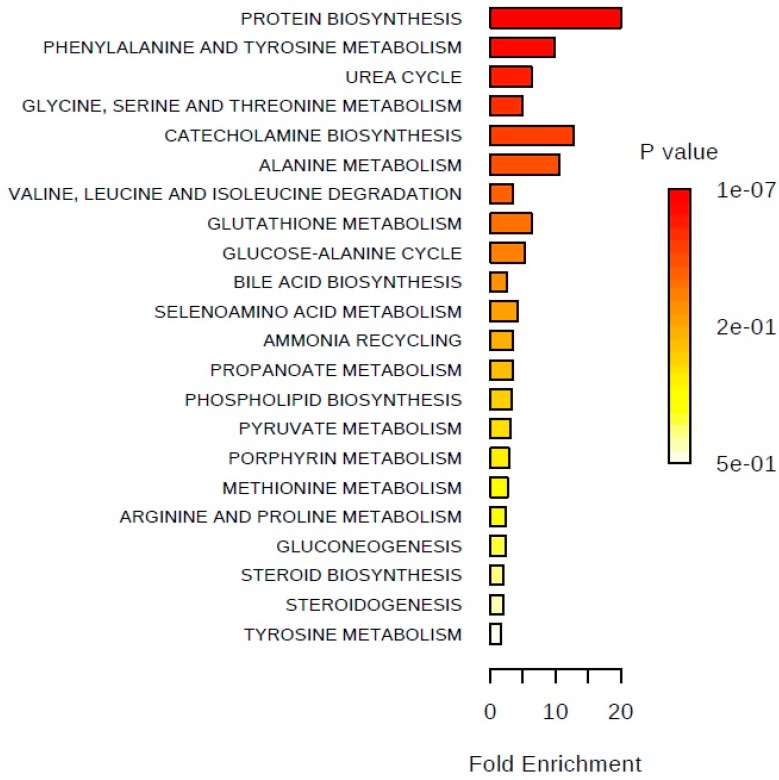
Metabolite sets enrichment overview; Summary plot for an over representation analysis (ORA) performed using all significantly altered metabolites between the normal prostate cell line (PNT2) and the PCa cell line with a higher metastatic potential (PC3).

To better understand the metabolic alterations associated with PCa, a metabolite set enrichment analysis (using MetaboAnalyst 3.0) [48] was also performed using all significantly altered metabolites found between the normal prostate cell line (PNT2) and the PCa cell line with the highest metastatic potential (PC3). The results revealed that the most significantly altered metabolic pathways in association with PCa were protein biosynthesis, phenylalanine and tyrosine metabolism, and the urea cycle. However, several other metabolic pathways were also altered in consequence of prostate carcinogenesis, such as the glucose-alanine cycle, phospholipid biosynthesis, and steroid biosynthesis (Figure 3). 

## 4. Materials and Methods

### 4.1. Chemicals

All the chemicals used in this study were of analytical grade. RPMI-1640 medium, phosphate buffered saline 1% (PBS), norvaline, methyl linolelaidate, desmosterol *N*-Methyl-*N*-(trimethylsilyl) trifluoroacetamide (MSTFA), and all analytical standards were purchased from Sigma-Aldrich Co. (St. Louis, MO, USA). The antibiotic mixture penicillin/streptomycin (10,000 U/mL of penicillin for every 10,000 µg/mL of streptomycin), heat inactivated fetal bovine serum (FBS), and trypsin 0.25%-EDTA were purchased from GIBCO Invitrogen (Barcelona, Spain). Sodium hydrogen carbonate was obtained from Merck (Darmstadt, Germany) and methanol was from VWR (Leuven, Belgium).

### 4.2. Cell Culture

PCa cell lines (PC3, 22RV1, DU145, and LNCaP) and the normal prostate epithelium immortalized cell line (PNT2) were provided by the Portuguese Oncology Institute of Porto (Supplementary Table S1). 22RV1 and LNCaP are cell lines from prostate carcinoma with AR expression, whereas PC3 and DU145 lack AR expression. Regarding the metastatic potential, PC3 is a cell line from grade IV adenocarcinoma with high metastatic potential, DU145 is a cell line from a prostate carcinoma with moderate metastatic potential, and LNCaP has low metastatic potential. All the cell lines were grown in RPMI-1640 medium supplemented with 10% of FBS and 1% of penicillin–streptomycin and were maintained at 37 °C under 5% CO_2_. 

### 4.3. Sample Collection

Four consecutive passages were used to evaluate the endometabolome. All cell lines were plated and grown to near confluence in RPMI-1640 medium. The cell culture medium was then rejected and the cells were washed with PBS solution twice. The PBS was also rejected and cells were carefully scraped with ice cold methanol, transferred to falcons on ice, and centrifuged at 3000× *g* for 10 min at 4 °C. Afterwards, the methanol with the extracted metabolites was separated from the pellet and immediately frozen at −80 °C until analysis. 

### 4.4. Metabolites Derivatization

Amino acids, fatty acids, sugars, and steroids cannot be analyzed directly by gas chromatography due to their low volatility and polarity and thus need to be derivatized prior to GC-MS analysis. The method used was previously developed by Pereira et al. (2012) with some adaptations. Briefly, the methanol with the extracted metabolites was centrifuged at 3000× *g* for 10 min at 4 °C, transferred to a glass vial, and the internal standards (10 µL/mL) (i.e., norvaline, methyl linolelaidate, and desmosterol) were added. Each sample (1 mL) was evaporated under a nitrogen stream and 50 µL of dichloromethane plus 50 µL of the derivatization reagent, MSTFA, were added to the residue. The vial was vortexed and heated for 30 min at 80 °C. Fifty microliters of the remaining residue was transferred to the glass vial used for GC-MS analysis [49]. All samples underwent the same procedure. 

### 4.5. GC-MS System and Data Acquisition

#### 4.5.1. GC-MS Analysis

The GC-MS conditions were based on those previously optimized by Pereira et al. (2012) with some adaptations. An EVOQ-436 gas chromatograph equipped with a Bruker Triple Quadrupole mass detector and a Bruker MS workstation software version 8.2 were used. The chromatographic separation was accomplished using a column Rxi-5Sil MS (30 m × 0.25 mm × 0.25 µm) (Restek). A CombiPAL automatic autosampler (Bruker) was used for all experiments. Sample extracts (1 μL) were injected using split mode (ratio 1/10). The carrier gas used was helium C-60 (Gasin, Portugal) (flow of 1 mL/min) and the injector port was heated to 250 °C. The analysis was performed in full scan mode. The oven temperature was fixed at 70 °C for 2 min, then increased to 250 °C (rate 15 °C/min), held for 2 min, and finally increased to 300 °C (rate 10 °C/min) and held for 8 min. The transfer line temperature was 250 °C and the manifold temperature was 40 °C. The mass range was from 50 to 1000 *m*/*z*. The emission current was 50 µA and the electron multiplier was set in relative mode to an auto tune procedure. All mass spectra were acquired in the electron impact (EI) mode [49]. All samples were randomly injected. 

To ensure reproducibility of the methodology, quality control samples (QCs) were injected [50] every six samples (four times per day). To produce these QCs, an equal volume of all samples (methanol extracts from the five cell lines) was used pooled, divided into several aliquots, and immediately frozen at −80 °C until analysis.

#### 4.5.2. Statistical Analysis

Prior to statistical analysis, all chromatograms were pre-processed with Mzmine 2.2 software, including baseline correction, peak detection, chromatogram deconvolution, and alignment [51]. The parameters used to pre-process the chromatograms were RT range (3.60–24.0 min), *m*/*z* range (50–500), MS data noise level (1.0 × 10^4^), *m*/*z* tolerance (0.3), chromatogram baseline level (2.0 × 10^4^), and peak duration range (0.02–0.30 min). All ions (*m*/*z*) with a RSD higher than 50% [50], as well as artefact ions (*m*/*z*) from the column, were removed. The data was normalized by the total area of the chromatograms and Pareto scaled. The multivariate analysis of the data included unsupervised (PCA) and supervised approaches (PLS-DA) used to evaluate the presence of relevant metabolic differences between the cell lines in the study. To confirm the robustness, all PLS-DA models were validated through a permutation test (200 random permutations of *Y*-observations and two components). To identify the compounds responsible for the discrimination of PCa cell lines from the normal cell line, the variable importance of projection (VIP) values obtained through PLS-DA were used. All metabolites with VIP values higher than one were considered potentially relevant for the separation of the studied cell lines. To confirm the importance of these metabolites in the discrimination between PCa cell lines and the normal prostate cell line and, consequently, their potential as PCa biomarkers, univariate analysis was performed using the Shapiro–Wilk test (to determine the normality distribution of data). To calculate the *p* value, the unpaired Student’s *t*-test with Welch correction (normal distribution) or the unpaired Mann–Whitney test (non-normal distribution) was used. The percentage of variation, uncertainty of the percentage of variation, effect size, and the standard error were calculated [52]. A Bonferroni correction [53] was used to adjust *p* values for multiple comparisons by setting the significance cut-off of the *p* value. After this univariate analysis, the metabolites taken into account were those with *p* < 0.05, a percentage of variation superior to the calculated uncertainty, and an effect size superior to the calculated standard error. For all these metabolites, the AUC value was calculated using MetaboAnalyst 3.0 [54]. Finally, the sensitivity and specificity of the sets of discriminant metabolites obtained for all comparisons were also calculated using MetaboAnalyst 3.0 [54]. 

The identification of compounds selected by statistical approaches was achieved by using the National Institute of Standards and Technology (NIST 14) mass spectral library and Kovat’s indices, and these were confirmed using the injection of authentic standards, when available (Supplementary Table S2).

## 5. Conclusions

In this study, we evaluated alterations in prostate cancer cells’ endometabolome using PCa cell lines with different aggressiveness and hormone-dependence status. The results showed that changes in the metabolomic profile are capable of discriminating cancer from normal prostate cells, enabling the discovery of new PCa biomarkers. 

Overall, we found alterations in the lipid, energy, and protein metabolisms of PCa cells. The dysregulations in these metabolic pathways might be explained by the well-known characteristics of cancer cells, namely their proliferative growth and migration capacity, as well as their resistance to apoptosis. To sustain these elevated proliferation rates, energy demands and protein synthesis increase. Lipid metabolism is involved in several cellular functions, like intercellular signaling, cell membrane synthesis, and energy production. The evaluation of alterations in these latter compounds (amino acids, sugars, fatty acids, and steroids) may allow for the discovery of novel biomarkers for PCa detection and a better understanding of the most important metabolic pathways altered because of cancer development and progression. This may lead to the discovery of new therapeutic targets and the development of new therapeutic approaches.

## Figures and Tables

**Table 1:** List of metabolites selected from PLS-DA of 22RV1 versus PNT2, PC3 versus PNT2, DU145 versus PNT2, and LNCaP versus PNT2 (VIP > 1) which are potentially important for discrimination between PCa and normal cell lines, and the results of univariate analysis. Values for *p* values, percentage of variation, effect size (ES), standard error (ESse), and area under the curve (AUC) are represented for each metabolite.

Chemical Name (IUPAC) or Common Name	22RV1 vs. PNT2				PC3 vs. PNT2				DU145 vs. PNT2				LNCaP vs. PNT2			
	*p* Value	Variation ± Uncertainty	ES ± ES_SE_	AUC	*p* Value	Variation ± Uncertainty	ES ± ES_SE_	AUC	*p* Value	Variation ± Uncertainty	ES ± ES_SE_	AUC	*p* Value	Variation ± Uncertainty	ES ± ES_SE_	AUC
Amino acids																
L-Alanine					0.0034	↓ 24.92 ± 8.48	↓ 1.18 ± 0.90	0.85	<0.0001 ^P^	↓ 53.75 ± 8.88	↓ 3.26 ± 1.20	0.98	0.0171	↓ 24.11 ± 9.88	↓ 1.13 ± 0.85	0.77
Glycine	0.0004 ^P^	↑ 335.89 ± 27.22	↑ 1.99 ± 0.98	0.90	0.0015	↑ 111.09 ± 27.01	↑ 1.35 ± 0.93	0.88	>0.05	↑	↑		0.0042	↑ 118.14 ± 20.13	↑ 1.57 ± 0.91	0.86
Sarcosine (*N*-methylglycine)					>0.05	↓	↓									
β-Alanine	<0.0001 ^P^	↓ 83.07 ± 21.15	↓ 2.52 ± 1.07	0.99	0.0066	↑ 78.49 ± 18.46	↑ 1.43 ± 0.94	0.84	<0.0001 ^P^	↑ 729.64 ± 20.99	↑ 2.95 ± 1.14	1	<0.0001 ^P^	↓ 87.83 ± 21.39	↓ 2.73 ± 1.12	1
L-Valine	<0.0001 ^P^	↓ 92.03 ± 12.16	↓ 5.20 ± 1.70	1	<0.0001 ^P^	↓ 62.12 ± 15.18	↓ 2.40 ± 1.10	0.97	<0.0001 ^P^	↓ 58.58 ± 11.82	↓ 2.76 ± 1.10	0.97	<0.0001 ^P^	↓ 71.40 ± 10.70	↓ 3.92 ± 1.38	1
L-Leucine	<0.0001 ^P^	↓ 72.24 ± 10.06	↓ 4.29 ± 1.47	1	0.0420	↓ 25.18 ± 12.60	↓ 1.00 ± 0.88	0.75	<0.0001 ^P^	↓ 45.52 ± 10.07	↓ 2.97 ± 1.16	0.95	<0.0001 ^P^	↓ 51.55 ± 9.06	↓ 2.97 ± 1.16	0.98
L-Proline	>0.05	↑ 21.11 ± 17.59	↑		>0.05	↑	↑		>0.05	↓	↓		>0.05	↓	↓	
L-Threonine	0.0187	↓ 37.40 ± 17.94	↓ 1.02 ± 0.84	0.79	0.0104	↓ 39.33 ± 16.31	↓ 1.18 ± 0.90	0.79	0.0499	↓ 26.44 ± 13.94	↓ 0.86 ± 0.81		0.0445	↓ 27.58 ± 14.13	↓ 0.88 ± 0.82	0.75
L-Aspartic acid	>0.05	↓	↓		>0.05	↑	↑		>0.05	↑	↑		>0.05	↓ 25.32 ± 22.20	↓	
L-Glutamine	<0.0001 ^P^	↑ 122.44 ± 13.48	↑ 2.36 ± 1.04	0.95	>0.05	↑ 35.65 ± 19.27	↑		>0.05	↓	↓		<0.0001 ^P^	↑ 108.67 ± 12.83	↑ 2.29 ± 1.03	0.92
Phenylalanine	0.0075	↑ 29.96 ± 8.45	↑ 1.23 ± 0.87	1	0.0056	↓ 35.65 ± 13.25	↓ 1.34 ± 0.92	0.86	0.0447	↓ 20.14 ± 10.71	↓		0.0036	↓ 42.45 ± 13.45	↓ 1.59 ± 0.91	0.85
L-Tyrosine	0.0002 ^P^	↑ 43.50 ± 7.52	↑ 1.92 ± 0.96	0.92	0.0322	↑ 27.81 ± 10.49	↑ 1.04 ± 0.89	0.73	0.0406	↓ 20.27 ± 8.09	↓ 0.90 ± 0.81	0.76	<0.0001 ^P^	↓ 44.38 ± 9.74	↓ 2.28 ± 1.03	0.93
Sugars																
Sorbose	<0.0001 ^P^	↓ 73.09 ± 10.30	↓ 4.22 ± 1.45	1	>0.05	↓	↓		0.0009 ^P^	↓ 37.57 ± 11.50	↓ 1.59 ± 0.89	0.86	<0.0001 ^P^	↓ 64.31 ± 10.68	↓ 3.41 ± 1.26	1
Organic acids derivatives																
Lactic Acid	<0.0001 ^P^	↓ 84.79 ± 7.44	↓ 7.43 ± 2.29	1	<0.0001 ^P^	↓ 31.78 ± 6.23	↓ 2.38 ± 1.10	0.96	0.0213	↓ 18.06 ± 7.62	↓ 1.03 ± 0.85	0.81	<0.0001 ^P^	↓ 55.39 ± 7.88	↓ 3.83 ± 1.36	1
3-Hydroxy-propionic acid									>0.05	↑	↑					
2-Butenoic acid	>0.05	↑ 29.61 ± 20.94	↑		>0.05	↑ 28.98 ± 11.45	↑		<0.0001 ^P^	↑ 130.36 ± 12.10	↑ 2.57 ± 1.06	0.99	>0.05	↑ 20.13 ± 14.41	↑	
3-Hydroxy-isovaleric acid					<0.0001 ^P^	↑ 802.11 ± 48.46	↑ 1.84 ± 1.00	1	<0.0001 ^P^	↑ 789.83 ± 18.65	↑ 3.37 ± 1.23	1	<0.0001 ^P^	↑ 3161.67 ± 34.43	↑ 2.40 ± 1.04	1
Toluic acid													>0.05	↓ 16.04 ± 10.59	↓	
Galacturonic acid	>0.05	↑ 115.65 ± 78.05	↑		>0.05	↑	↑		>0.05	↑	↑		>0.05	↑	↑	
Fatty acids																
Tridecanoic acid					>0.05	↑	↑		>0.05	↑ 63.28 ± 58.62	↑					
Palmitic Acid	>0.05	↓ 13.54 ± 6.80	↓ 0.86 ± 0.83		>0.05	↑	↑		>0.05	↑	↑		0.0424	↑ 21.27 ± 8.34	↑ 0.95 ± 0.84	0.78
9-Hexadecenoic acid (palmitoleic acid)	<0.0001 ^P^	↑ 284.2 ± 18.01	↑ 2.81 ± 1.13	1					0.0048	↑ 85.75 ± 23.12	↑ 1.02 ± 0.83	0.83				
13-Octadecenoic acid													<0.0001 ^P^	↑ 560.66 ± 18.06	↑ 3.54 ± 1.29	1
Steroids																
Cholesterol	0.0004 ^P^	↑ 110.4 ± 15.40	↑ 1.96 ± 0.97	0.91	<0.0001 ^P^	↑ 65.91 ± 7.57	↑ 3.01 ± 1.23	0.99	>0.05	↑ 20.85 ± 12.10	↑		0.0004 ^P^	↑ 73.42 ± 14.39	↑ 1.58 ± 0.91	0.91
Others																
Ethanolamine	<0.0001 ^P^	↓ 97.18 ± 18.37	↓ 3.80 ± 1.35	1	0.0052	↓ 54.13 ± 21.78	↓ 1.41 ± 0.93	0.83	<0.0001 ^P^	↓ 80.14 ± 18.04	↓ 2.92 ± 1.31	0.99	<0.0001 ^P^	↓ 89.19 ± 17.26	↓ 3.47 ± 1.28	1
Urea	0.0002 ^P^	↓ 36.52 ± 9.04	↓ 1.93 ± 0.97	0.92	<0.0001^p^	↓ 45.10 ± 9.41	↓ 2.36 ± 1.09	0.97	<0.0001 ^P^	↓ 47.59 ± 9.66	↓ 2.31 ± 1.01	0.97	>0.05	↑ 8.39 ± 7.20	↑	
Glycerol	>0.05	↑ 32.43 ± 19.85	↑						0.0005 ^P^	↓ 60.06 ± 18.39	↓ 1.84 ± 0.93	0.90	0.0004 ^P^	↓ 58.18 ± 15.77	↓ 1.97 ± 0.97	0.98
Creatinine									>0.05	↓ 51.11 ± 46.5	↓					
Methyl 2-acetamido-2-deoxy-3-*O*-methyl-a-d-galactopyrano-side					>0.05	↑ 196.92 ± 50.03	↑ 1.05 ± 0.89		0.0037	↑ 88.07 ± 16.92	↑ 1.42 ± 0.87	0.82	0.0050	↑ 374.03 ± 5.88	↑ 1.57 ± 0.91	0.89
Unknowns																
Unknown 1					>0.05	↑ 69.09 ± 27.41	↑ 0.90 ± 0.87						0.0001 ^P^	↓ 70.35 ± 22.44	↓ 1.82 ± 0.94	0.94
Unknown 2	0.0309	↑ 55.75 ± 17.47	↑ 1.04 ± 0.84	0.71	0.0006 ^P^	↓ 44.67 ± 12.70	↓ 1.67 ± 0.97	0.69	<0.0001 ^P^	↓ 56.86 ± 15.31	↓ 2.05 ± 0.97	0.92	0.0321	↑ 93.47 ± 25.21	↑ 1.07 ± 0.84	0.70
Unknown 3	>0.05	↓ 54.68 ± 39.46	↓		>0.05	↓ 37.52 ± 36.65	↓		>0.05	↓	↓		>0.05	↓ 40.90 ± 32.77	↓	
Unknown 4					<0.0001 ^P^	↑ 182.71 ± 16.77	↑ 2.97 ± 1.22	1	0.0018	↑ 82.93 ± 11.28	↑ 2.05 ± 0.97	0.86	<0.0001 ^P^	↑ 64.27 ± 7.16	↑ 2.80 ± 1.13	0.95
Unknown 5									>0.05	↑ 47.15 ± 41.17	↑					
Unknown 6	<0.0001 ^P^	↑ 222.97 ± 14.87	↑ 3.03 ± 1.17	1	0.0008 ^P^	↑ 132.53 ± 18.38	↑ 2.18 ± 1.06	0.95	0.0078	↑ 70.10 ± 16.23	↑ 1.26 ± 0.85	0.83				
Unknown 7	>0.05	↓	↓						>0.05	↑ 63.22 ± 58.62	↑					
Unknown 8									0.0002 ^P^	↑ 71.71 ± 10.89	↑ 1.91 ± 0.94	0.93				
Unknown 9	0.0106	↑ 25.74 ± 8.04	↑ 1.16 ± 0.85	0.81	>0.05	↓ 16.43 ± 9.53	↓		0.0014	↑ 27.91 ± 7.39	↑ 1.31 ± 0.87	0.87	>0.05	↑ 15.5 ± 7.07	↑	
Unknown 10	0.0252	↑ 24.74 ± 8.62	↑ 1.04 ± 0.84	0.79	0.0032	↓ 29.52 ± 9.79	↓ 1.48 ± 0.94	0.90	>0.05	↑ 11.09 ± 7.51	↑		0.0256	↑ 20.39 ± 7.37	↑ 1.02 ± 0.84	0.76
Unknown 11													<0.0001 ^P^	↑ 147.83 ± 10.00	↑ 3.53 ± 1.29	1
Unknown 12													>0.05	↑ 25.39 ± 16.87	↑	
Unknown 13					<0.0001 ^P^	↓ 56.54 ± 9.08	↓ 3.52 ± 1.35	0.99								
Unknown 14									>0.05	↑	↑					
Unknown									<0.0001 ^P^	↑ 326.66 ± 19.57	↑ 2.50 ± 1.04	0.96				

↑ Represents metabolites increased and ↓ represents metabolites decreased in PCa cells compared with normal cell line. ^P^ Alterations remaining significant after Bonferroni correction, with cutoff *p*-value of 1.92 × 10^−3^ (0.05 divided by 26 analyzed metabolites), for 22RV1 versus PNT2; 1.61 × 10^−3^ (0.05 divided by 31 analyzed metabolites) for PC3 versus PNT2; 1.35 × 10^−3^ (0.05 divided by 37 analyzed metabolites) for DU145 versus PNT2; and 1.56 × 10^−3^ (0.05 divided by 32 analyzed metabolites) for LNCaP versus PNT2. At bold are represented the results that are statistically significant.

**Table 2:** Sensitivity and specificity of the discriminant sets obtained.

	Sensitivity	Specificity
22RV1 vs. PNT2	100%	100%
PC3 vs. PNT2	100%	100%
DU145 vs. PNT2	100%	100%
LNCaP vs. PNT2	97%	100%

## Supplementary Materials

The following are available online at http://www.mdpi.com/2218-1989/8/1/23/s1.
