# Supplementary material for: GC-MS-Based Endometabolome Analysis Differentiates Prostate Cancer from Normal Prostate Cells
Source: Metabolites. 2018 Mar 19;8(1):23. doi: 10.3390/metabo8010023 (PMC5876012; doi:10.3390/metabo8010023)
Supplement: Supplementary file 1 [file metabolites-08-00023-s001.pdf]

GC-MS-based endometabolome analysis differentiates prostate cancer from normal prostate cells

Ana Rita Lima[1], Ana Margarida Araújo[1], Joana Pinto[1], Carmen Jerónimo[2,3], Rui Henrique[2,3,4], Maria de Lourdes Bastos[1], Márcia Carvalho[1,5], Paula Guedes de Pinho[1]

[1]UCIBIO/REQUIMTE, Department of Biological Sciences, Laboratory of Toxicology, Faculty of Pharmacy, University of Porto, Porto, Portugal

[2]Cancer Biology & Epigenetics Group, Research Center (CI-IPOP) Portuguese Oncology Institute of Porto (IPO Porto), Porto, Portugal

[3]Department of Pathology and Molecular Immunology-Biomedical Sciences Institute (ICBAS), University of Porto, Porto, Portugal

[4]Department of Pathology, Portuguese Oncology Institute of Porto (IPO Porto), Porto, Portugal

[5]UFP Energy, Environment and Health Research Unit (FP-ENAS), University Fernando Pessoa, Porto, Portugal

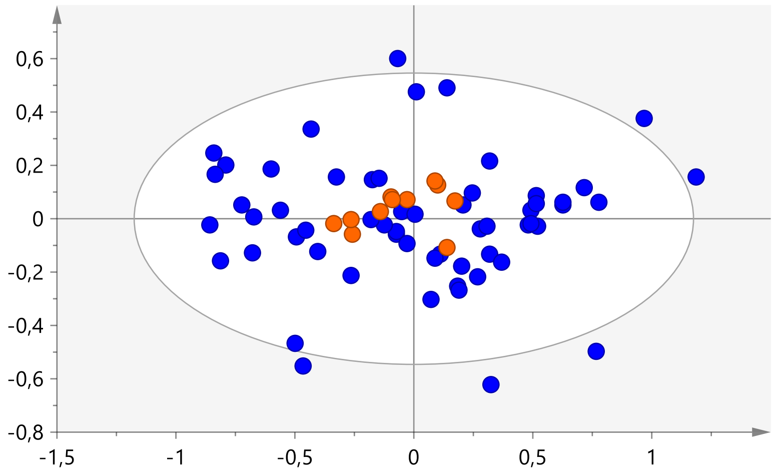

Supplementary Fig. 1: PCA score scatter plot obtained for the GC-MS chromatograms of all samples, namely QCs samples (in orange), and the of all cell lines and blanks (blue) (R^2X = 0.652).

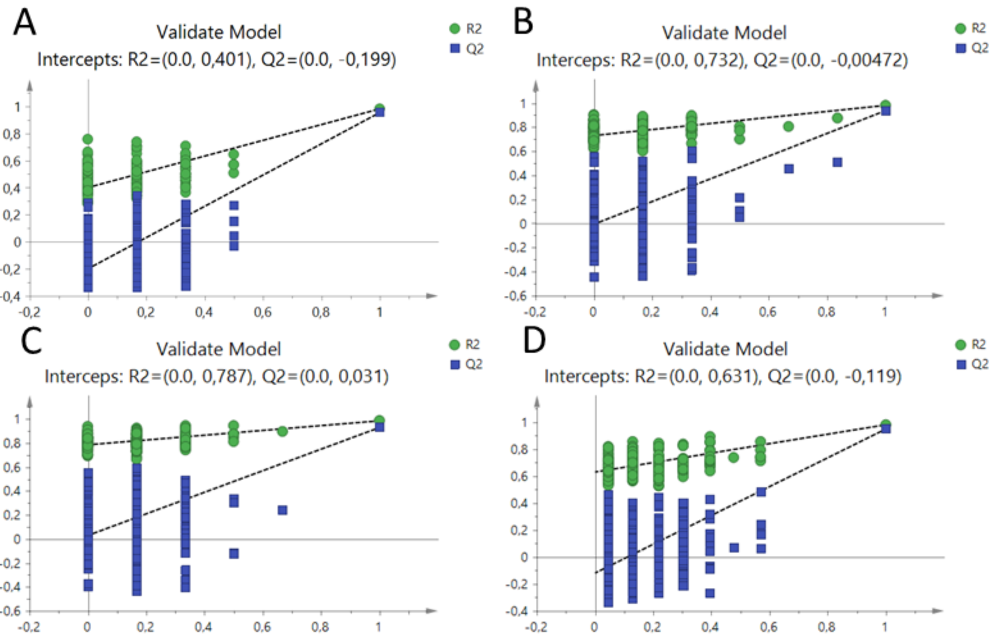

Supplementary Fig 2: Statistical validation of the PLS-DA models by permutation testing (200 permutations) (A: 22RV1 vs PNT2; B: PC3 vs PNT2; C: DU145 vs PNT2; D: LNCaP vs PNT2).

Supplementary Table 1: Characteristics of prostate cell lines used in this study.

	PNT2	22RV1	PC3	DU145	LNCaP
**Organism**	*Homo sapiens*	*Homo sapiens*	*Homo sapiens*	*Homo sapiens*	*Homo sapiens*
**Age**	33 years	NA	62 years	69 years	50 years
**Ethnicity**	NA	NA	Caucasian	Caucasian	Caucasian
**Tissue**	Prostate	Prostate	Prostate; derived from metastatic site: bone	Prostate; derived from metastatic site: brain	Prostate; derived from metastatic site: left supraclavicular lymph node
**Morphology**	Epithelial	Epithelial	Epithelial	Epithelial	Epithelial
**Culture Properties**	Adherent	Adherent	Adherent	Adherent	Adherent
**Disease**	Healthy	Carcinoma	Grade IV, adenocarcinoma	Carcinoma	Carcinoma
**Tumorigenic**	No	Yes	Yes	Yes	Yes
**AR expression**	Yes	Yes	No	No	Yes
**Metastatic potential**	—	NA	High	Moderate	Low

NA: not available

Supplementary Table 2: List of metabolites selected from PLS-DA of 22RV1 vs PNT2, PC3 vs PNT2, DU145 vs PNT2 and LNCaP vs PNT2 (VIP>1) as potentially important for discrimination between PCa and normal cell lines. The identification of the metabolites is based on the NIST (2014) and standards. They are characterized by their IUPAC name, RT, characteristic ions, Kovat indices from literature, experimental Kovat indices, NIST R-match, Cas registry number and HMDB code (when available).

Name	RT	Characteristic ions	KI from literature	Experimental KI or standards	MS-R match	Cas number	HMDB
**Unknown 1**	**4.56**	**69/140**	**NA**	**983**	**NA**	**NA**	**NA**
**Ethanolamine,** 2TMS derivative	**5.08**	**102/147**	**1021**	**1027**	**890**	**17165-52-5**	**HMDB00149**
**Lactic Acid,** 2TMS derivative*	**5.43**	**73/147**	**1066**	**1057**	**927**	**17596-96-2**	**HMDB00190**
**L-Alanine,** 2TMS derivative	**5.94**	**116/147/190**	**—**	**S**	**935**	**27844-07-1**	**HMDB00161**
**Glycine,** 2TMS derivative	**6.14**	**102/147/204**	**—**	**S**	**950**	**7364-42-3**	**HMDB00123**
**Sarcosine,** 2TMS derivative	**6.32**	**73**	**1161**	**S**	**845**	**7364-43-4**	**HMDB00271**
**3-Hydroxypropionic acid** 2TMS derivative	**6.41**	**127**	**1151**	**1140**	**842**	**55162-32-8**	**HMDB00700**
**ß-Alanine,** 2TMS derivative	**6.90**	**102/176**	**1190**	**1186**	**899**	**17891-86-0**	**HMDB00056**
**2-Butenoic acid, 2-[(trimethylsilyl)oxy]-trimethylsilyl ester**	**6.96**	**73/147**	**1186**	**1192**	**793**	**55590-70-0**	**HMDB10720**
**3-Hydroxyisovaleric acid,** 2TMS derivative	**7.10**	**73/131**	**1216**	**1202**	**937**	**55124-90-8**	**HMDB00754**
**L-Valine,** 2TMS derivative	**7.17**	**144/218**	**—**	**S**	**920**	**7364-44-5**	**HMDB00883**
**Urea,** 2TMS derivative	**7.43**	**73/147**	**1249**	**1219**	**921**	**18297-63-7**	**HMDB00294**
**L-Leucine,** 2TMS derivative	**7.94**	**102/158**	**—**	**S**	**931**	**7364-46-7**	**HMDB00687**
**L-Proline,** 2TMS derivative	**8.01**	**73/142/216**	**—**	**S**	**909**	**7364-47-8**	**HMDB00162**
**Unknown 2**	**8.54**	**147/204**	**NA**	**1277**	**NA**	**NA**	**NA**
**Toluic acid,** TMS derivative	**8.64**	**65/119/193**	**1282**	**1282**	**818**	**NA**	**HMDB62810**
**L-Threonine,** 2TMS derivative	**8.77**	**73/117/130**	**—**	**S**	**737**	**7536-82-5**	**HMDB00167**
**Glycerol,** 3TMS derivative	**8.92**	**103/147/205**	**1289**	**1297**	**789**	**6787-10-6**	**HMDB00131**
**L-Aspartic acid,** 3TMS derivative	**9.93**	**100**	**—**	**S**	**890**	**55268-53-6**	**HMDB00191**
**Creatinine, N,N,O-tris(trimethylsilyl)**	**10.26**	**75**	**1445**	**1447**	**758**	**NA**	**HMDB00562**
**L- Glutamine,** 3TMS derivative	**10.73**	**73/75/246**	**—**	**S**	**570**	**70591-28-5**	**HMDB00641**
**Phenylalanine,** 2TMS derivative	**10.84**	**73/147/218**	**—**	**S**	**876**	**2899-52-7**	**HMDB00159**
**Unknown 3**	**11.77**	**73**	**NA**	**1646**	**NA**	**NA**	**NA**
**Tridecanoic acid,** TMS derivative	**12.43**	**117**	**1705**	**1739**	**688**	**169597-14-2**	**HMDB00910**
**Sorbose,** 5TMS derivative	**13.02**	**103/217**	**1867**	**1826**	**809**	**NA**	**HMDB01266**
**L-Tyrosine,** 3TMS derivative	**13.06**	**73/100/218/280**	**—**	**S**	**923**	**51220-73-6**	**HMDB00158**
**Unknown 4**	**13.61**	**73/117/129**	**NA**	**1904**	**NA**	**NA**	**NA**
**Palmitic Acid,** TMS derivative	**13.77**	**73/132/129/313**	**—**	**S**	**935**	**55520-89-3**	**HMDB00220**
**Galacturonic acid,** 5TMS derivative	**14.04**	**75**	**1943**	**1924**	**729**	**NA**	**HMDB02545**
**Unknown 5**	**14.21**	**131**	**NA**	**1932**	**NA**	**NA**	**NA**
**Unknown 6**	**14.25**	**73/145/311**	**NA**	**1934**	**NA**	**NA**	**NA**
**Unknown 7**	**14.42**	**117**	**NA**	**1942**	**NA**	**NA**	**NA**
**Unknown 8**	**14.47**	**117/327**	**NA**	**1944**	**NA**	**NA**	**NA**

Name	RT	Characteristic ions	KI from literature	Experimental KI or standards	MS-R match	Cas number	HMDB
**Unknown 9**	14.94	117/129/339	NA	1966	NA	NA	NA
**Unknown 10**	15.14	117/129/145/341	NA	1975	NA	NA	NA
**Unknown 11**	15.62	57/69/84/	NA	1997	NA	NA	NA
**9-Hexadecenoic acid,** TMS derivative **(palmitoleic acid)**	15.80	55/75/145	2027	2006	701	NA	HMDB03229
**Unknown 12**	16.28	117	NA	2028	NA	NA	NA
**Unknown 13**	16.78	75/129	NA	2051	NA	NA	NA
**Unknown 14**	17.30	55	NA	2075	NA	NA	NA
**Methyl 2-acetamido-2-deoxy-3-O-methyl-a-D-galactopyranoside,** 2TMS	17.94	73/117	2134	2105	660	56196-89-5	NA
**Unknown 15**	19.00	73/117	NA	2154	NA	NA	NA
**13-Octadecenoic acid,** TMS derivative	19.72	69/75/117	2228	2187	634	NA	HMDB41480
**Cholesterol,** TMS derivative	23.06	129/329/368	—	S	912	1856-05-9	HMDB00067

KI: Kovat indices; HMDB: Human Metabolome Database; NA: not available; S: identified with standards;
